# The associations between smoking and obesity in northeast China: a quantile regression analysis

**DOI:** 10.1038/s41598-019-39425-6

**Published:** 2019-03-14

**Authors:** Mengzi Sun, Yan Jiang, Chong Sun, Jiagen Li, Xin Guo, Yaogai Lv, Yaqin Yu, Yan Yao, Lina Jin

**Affiliations:** 1 Department of Epidemiology and Biostatistics, School of Public Health, Jilin University, Changchun, Jilin China; 2 Department of Biostatistics, Graduate School of Public Health, University of Pittsburgh, Pittsburgh, PA USA

## Abstract

Obesity is a risk factors of chronic diseases, and smoking is associated with both chronic diseases and obesity. There were some controversies about the associations between smoking and obesity. Thus, our study aimed to explore the associations of smoking with obesity, using body mass index (BMI) and waist circumference (WC) as obesity indices in northeast China. We enrolled a sample of 16,412 participants in Jilin province aged 18–79 in this study, which were derived from a cross-sectional survey in 2012. We used quantile regression (QR) models to identify the associations of smoking with obesity in different quantiles of BMI (or WC) by genders. The differences of BMI and WC by genders were statistically significant (*p* < 0.05). In conclusion, compared with current non-smokers, current smokers had lower BMI but higher WC. As increasing of WC, the association of WC with smoking was getting stronger, especially in females.

## Introduction

Obesity has become a serious public health crisis worldwide, and there was a large increase in the number of Chinese adults with overweight and obesity over the last two decades^1–4^. In 2010, in Chinese adults over the 18 years old, the prevalence of overweight and obesity was 27.9% and 5.1% respectively^4^. Obesity is also a major risk factor of many chronic diseases, such as cardiovascular diseases (CVD), hypertension, hyperlipidemia and certain types of cancer^5–7^. Currently, a variety of indices have been adopted to evaluate the obesity status in some studies^8–10^, like body mass index (BMI), waist circumference (WC), waist-hip ratio (WHR) and waist-to-height ratio (WHtR). In addition, it was reported that different populations had different optimal obesity indices^11^.

BMI is one of the most commonly used indices to define general obesity, and WC is widely used to define abdominal obesity^9,12^. However, BMI cannot distinguish between lean mass and fat mass, and WC cannot account for the influence of height on risk^13^. It was supported that BMI and WC were the optimal combinations for Chinese people to predict CVD risk factors and metabolic syndrome^14^.

There was compelling evidence implicating that obesity was affected by varieties of factors, where smoking was one of them^15–17^. The Chinese smoking rate was 28.1% in 2010 by the China Global Adults Tobacco Survey^18^. It was estimated that, in China, 2 million smokers would die each year due to smoking-related diseases by 2020^19^. Some studies also investigated the combined effect of smoking and obesity, with a notable higher risk of chronic disease in obese smokers^20,21^. Interestingly, numerous studies demonstrated that smokers tended to have lower BMI than non-smokers^22,23^, however, a cross-sectional study indicated that WC was higher in smokers compared with non-smokers^24^. There were several studies focused on the smoking-abdominal obesity and general obesity associations among adults in the Chinese population. Moreover, previous studies divided BMI into groups as categorical variables, which may lead to neglect of useful information and could not get the precise relationship between smoking and obesity.

Although the associations between obesity and smoking have been extensively investigated, there are few studies examined the effects of smoking on combinations of BMI and WC. In addition, the prevalence of overweight, obesity and smoking in Jilin Province was 32.3%, 14.6% and 39.1%, respectively, which were extremely higher than the Chinese average level^25–27^. The aims of this study were to explore the associations between smoking and obesity in different quantiles of BMI and WC among the population in Jilin Province, China, using quantile regression (QR) model. We tested the different associations of smoking on WC via adjusting and un-adjusting BMI as confounders.

## Results

### Descriptive Characteristics of Participants by Gender

A total of 16,412 participants (median age was 48.00 years old, including 7,517 males and 8,895 females (54.20%)) were enrolled in this study, and baseline characteristics were presented in Table 1. There were significant differences between males and females in the distributions of educational level, smoking status, drinking status, dietary habits and physical exercise, respectively (*p* < 0.05), and females were older than males (*p* < 0.05). Table 2 showed the distribution of BMI and WC by genders and by smoking status, and the differences were statistically significant (*p* < 0.05). The different quantiles of WC in different BMI groups by genders were described in the Supplemental Materials (Table S1 and S2).

**Table 1:** Descriptive characteristics of participants by gender (M [95% CI]/n (%) [95% CI]).

Variables	Male		Female		t/χ^2^	*p*-value
	n	Mean/% [95% CI]	n	Mean/% [95% CI]		
Age(years)^a^		43.25 [42.83, 43.67]		43.98 [43.55, 44.42]	281.967	<0.001
BMI		24.30 [24.20, 24.41]		23.83 [23.73, 23.93]	644.045	<0.001
WC		84.33 [84.03, 84.63]		78.89 [78.62, 79.16]	768.330	<0.001
WHtR(10^−3^)		496.73 [494.99, 498.46]		500.10 [498.29, 501.90]	702.820	<0.001
Urban^b^					0.194	0.718
City	3944	55.22 [53.88, 56.55]	4569	54.88 [53.58, 56.16]		
Country	3573	44.78 [43.45, 46.12]	4326	45.12 [43.84, 46.42]		
Educational level^b^					248.698	<0.001
Primary school and below	1624	19.02 [18.00, 20.07]	3185	29.14 [28.07, 30.23]		
Middle school	2271	30.44 [29.20, 31.70]	2422	29.37 [28.15, 30.62]		
High school	2237	29.89 [28.69, 31.13]	2083	24.26 [23.20, 25.35]		
College degree and above	1385	20.65 [19.56, 21.79]	1205	17.23 [16.19, 18.33]		
Smoking status^b^					3710.181	<0.001
Yes	3950	52.86 [51.51, 54.20]	1013	9.09 [8.46, 9.77]		
No	3567	47.14 [45.80, 48.49]	7882	90.91 [90.23, 91.54]		
Drinking status^b^					3923.116	<0.001
Yes	4316	56.74 [55.39, 58.09]	843	10.70 [9.87, 11.59]		
No	3201	43.26 [41.91, 44.61]	8052	89.30 [88.41, 90.13]		
Dietary habits^b^					718.179	<0.001
Balance	4755	64.73 [63.46, 65.98]	4940	58.26 [56.99, 59.51]		
Meat more	976	13.44 [12.53, 14.40]	384	4.55 [4.04, 5.12]		
Vegetable more	1786	21.83 [20.80, 22.90]	3571	37.19 [35.97, 38.43]		
Physical exercise^b^					19.555	0.002
Often	2264	28.26 [27.04, 29.50]	2575	25.65 [24.62, 26.72]		
Sometimes	1881	28.43 [27.19, 29.70]	2078	27.92 [26.68, 29.19]		
Never/Rare	3372	43.31 [42.00, 44.64]	4242	46.43 [45.15, 47.72]		

^a^was described by mean [95% CI], ^b^was described by n (%) [95% CI].

**Table 2:** The distribution of BMI and WC by genders and smoking status (M [95% CI]/n (%) [95% CI]).

Variables	Male				Female			
	Smoking	Non-smoking	t/χ^2^	*P*	Smoking	Non-smoking	t/χ^2^	*P*
BMI groups^a^			89.311	<0.001			15.401	0.014
Underweight	175 (5.33) [4.27, 6.63]	102 (3.53) [2.78, 4.46]			88 (9.46) [7.04, 12.59]	325 (5.98) [5.22, 6.85]		
Normal	1946 (48.25) [46.37, 50.14]	1401 (39.79) [37.93, 41.68]			485 (47.96) [44.29, 51.66]	3619 (49.06) [47.68, 50.43]		
Overweight	1321 (33.10) [31.33, 34.92]	1420 (38.19) [36.36, 40.06]			318 (30.40) [27.26, 33.74]	2772 (31.61) [30.41, 32.84]		
Obese	508 (13.32) [12.12, 14.62]	644 (18.49) [17.04, 20.03]			122 (12.18) [9.91, 14.88]	1166 (13.35) [12.51, 14.24]		
BMI	23.88 [23.73, 24.03]	24.78 [24.63, 24.93]	448.152	<0.001	23.47 [23.16, 23.78]	23.86 [23.76, 23.97]	467.022	<0.001
WC groups^b^			35.291	<0.001			6.147	0.023
Normal	2159 (54.76) [52.88, 56.64]	1631 (47.90) [45.99, 49.83]			484 (50.76) [47.06, 54.44]	3852 (55.31) [53.96, 56.64]		
Obese	1791 (45.24) [43.36, 47.12]	1936 (52.10) [50.17, 54.01]			529 (49.24) [45.56, 52.94]	4030 (44.69) [43.36, 46.04]		
WC	83.43 [83.02, 83.85]	85.33 [84.91, 85.75]	558.421	<0.001	79.93 [79.17, 80.69]	78.79 [78.50, 79.08]	573.404	<0.001
WHtR(10^−3^)	490.21 [487.84, 492.58]	504.04 [501.53, 506.55]	505.632	<0.001	510.80 [505.84, 515.76]	499.02 [497.11, 500.09]	489.118	<0.001

^a^Underweight: BMI < 18.5 kg/m^2^; Normal: 18.5 ≤ BMI < 24.0 kg/m^2^; Overweight: 24.0 ≤ BMI < 28.0 kg/m^2^; Obese: BMI ≥ 28.0 kg/m^2^.

^b^WC ≥ 85 cm for males and WC ≥ 80 cm for females were defined as obese.

### The Results of QR Models

We listed the dependent and independent variables of QR models (Table 3), and the coefficients of smoking associated with BMI and WC in QR models by genders (Table 4). Model 1 showed that smoking was negatively associated with BMI, and Model 2 showed that smoking was also negatively associated with WC. However, smoking was positively associated with WC after BMI adjustment in Model 3 and the associations of smoking with obesity were different by gender; for females, only when WC was bigger than its 50% level, the associations were significant. As quantiles of BMI (or WC) increasing, the coefficients of smoking were getting larger in almost all quantiles, especially in females. The details of the coefficients of other variables in QR models were listed in the Supplemental Materials (Table S3~S8).

**Table 3:** Information of QR models.

	Dependent variable	Independent variables
Model 1	BMI	Age + urban + educational level + occupation + marital status + smoking status + drinking status + dietary habits + physical exercise
Model 2	WC	Age + urban + educational level + occupation + marital status + smoking status + drinking status + dietary habits + physical exercise
Model 3	WC	Age + urban + educational level + occupation + marital status + smoking status + drinking status + dietary habits + physical exercise + BMI

**Table 4:** Coefficients (p-value) of current smokers comparing with current non-smokers in QR models by genders.

Models	P_10_	P_20_	P_30_	P_50_	P_70_	P_85_	P_95_
Male							
Model 1	−0.905 (<0.001)	−0.856 (<0.001)	−0.971 (<0.001)	−0.928 (<0.001)	−0.774 (<0.001)	−1.005 (<0.001)	−0.970 (<0.001)
Model 2	−1.836 (<0.001)	−2.071 (<0.001)	−1.907 (<0.001)	−1.513 (<0.001)	−1.573 (<0.001)	−1.540 (0.005)	−1.609 (0.015)
Model 3	0.547 (0.015)	0.646 (<0.001)	0.603 (<0.001)	0.624 (<0.001)	0.325 (0.080)	0.480 (0.028)	1.069 (<0.001)
Female							
Model 1	−1.751 (<0.001)	−1.596 (<0.001)	−1.400 (<0.001)	−1.170 (<0.001)	−0.808 (<0.001)	−0.634 (<0.001)	−0.211 (0.670)
Model 2	−2.765 (<0.001)	−3.223 (<0.001)	−2.458 (<0.001)	−2.000 (0.002)	−1.000 (0.153)	−0.315 (0.650)	0.109 (0.912)
Model 3	0.475 (0.114)	0.413 (0.112)	0.504 (0.091)	0.738 (0.006)	0.976 (<0.001)	1.039 (0.003)	0.957 (0.028)

## Discussion

Although the associations between obesity and smoking have been extensively investigated, there are few studies examined the effects of smoking on combinations of BMI and WC. In this study, QR models were adopted to explore detailed associations between smoking and obesity by BMI and WC, the results showed that current smokers had a lower BMI but a higher WC than current non-smokers; and as WC increasing, the association between WC and smoking was getting stronger, especially in females.

Compelling evidence implicated that smokers tended to have lower BMI than non-smokers^22^, which consisted with our results. This might because nicotine could suppress appetite by acting on brain and extend inter-meal interval, thus less food intake resulted in weight loss^28,29^. The China Kadoorie biobank study showed that WC was negatively associated with smoking without BMI adjustment while WC was positively associated with smoking after adjusting for BMI^22^, which was consistent with our study. Moreover, presented by QR model in our study, the strength of the associations of WC with smoking was getting stronger as WC increasing, which might imply that smoking would have a stronger impact on the people with larger WC than those people with smaller WC. The reasons for this might be: (i) Smoking may be associated with increased insulin resistance by reducing insulin sensitivity or stimulating the production of hyperglycemic hormones (mainly epinephrine and norepinephrine)^30,31^. And insulin resistance was known to be associated with increased in abdominal fat deposition, which might be caused by inefficient decomposition of subcutaneous fat^32^; (ii) The genetic disposition to smoking might lead to WC increasing, via the minor allele associated with cigarette consumption^33^. This might indicate that smoking could lead to central adiposity, although weight loss was at the level of appearances. Those with bigger waists should pay more attention of effect of smoking on WC.

Furthermore, compared with males, the associations of obesity and smoking were stronger in females, especially when WC was bigger than its 50% level. Our findings were similar with previous evidence^34^, and in Scotland being a female smoker was associated with greater central adiposity^35^. This may due to the strong anti-estrogenic effect of nicotine^36^. Therefore, intervention in smoking should be strengthened, especially for females with bigger waists.

Our study had some limitations. Firstly, our study was a cross-sectional study in Jilin Province, which might limit the ability to generalize the results. Secondly, the health-related behaviors and dietary habits of participants were based on self-report, which may be subject to reporting bias. Thirdly, among current smokers, there were more males than females, which may lead to insufficient convincing conclusions. Fourthly, not all indexes used to evaluate obesity, such as WHtR was included in this study. Lastly, we could not consider other confounders (i.e., genes), which might have slight impacts on the associations of obesity and smoking.

## Conclusions

Compared with current non-smokers, current smokers had lower BMI but higher WC; and as WC increasing, the association of WC with smoking was getting stronger, especially in females.

## Methods

### Study Population

Data was derived from a cross-sectional study of chronic disease conducted by School of Public Health, Jilin University and the Jilin Department of Health in Jilin Province of China in 2012. The planning sample size of this cross-sectional study was 23,050. During the investigation, 2,190 subjects were performed a replacement due to the invalid response sample. Finally, a total of 21,435 self-reported effective response participants were selected through multistage stratified random cluster sampling and the effective response rate was 84.9%. Those people had lived in Jilin Province for more than 6 months and were 18~79 years old. For the purpose of the present analyses, we excluded some subjects due to having missing values (4,560 subjects) and having limitation to do independent activities (463 subjects). Finally, a total of 16,412 subjects were included in the study. The detail information of study population was in the Supplementary Materials.

### Data Collection and Measurement

The data collection included an intensive investigation and household survey. The details of data collection were presented in the Supplementary Materials. All information was collected by investigators who had been uniformly trained. The data included demographics (i.e., gender, age), health-related behaviors (i.e., smoking status, drinking status) and anthropometric measurements (i.e., height, weight). Current smokers were defined as who had smoked at least 100 cigarettes lifetime. Otherwise we considered individuals as current non-smokers (that includes never-smokers)^37^. Participants who drank alcoholic beverages at least once a week in the past one year were characterized as drinkers. We classified educational level into four groups: primary school and below (including those who had never attended school and those with elementary schooling only); middle school (including those who were received 7–9 years education); high school (including those who were received 10–12 years education); college degree and above (including those who were received more than 13 years education). We categorized occupation into 3 groups: manual labor (who worked in the manufacturing industry, agriculture, and service industry), mental labor (who were managers, administrators, and technical personnel) and retirees or unemployed. Community was divided into two groups: urban (including those who lived in cities or towns) and rural (including the rest of the entire population). Marital status was divided into four groups: married (including those who were married and those co-habiting), never married, divorced or separated, and widowed. Physical exercises were classified into 3 categories according to last one year’s situation: often exercise (who engaged in physical activity at least 3 times a week), sometimes exercise (who engaged in physical activity once or twice a week) and never or rare exercise (who never or rare engaged in physical exercise). The participants’ height and weight were measured according to a standardized protocol with the participants wearing clothing but no shoes, and WC was measured at the midpoint between the lowest rib margin and the iliac crest with the participants standing. BMI and WHtR were calculated using the following formula: BMI = Weight (kg)/Height^2^ (m); WHtR = WC (cm)/Height (cm). In terms of abdominal obesity status, the Chinese criteria were used to classify individuals as obese (WC ≥ 85 cm for males and WC ≥ 80 cm for females) and normal. BMI was divided into 4 groups of underweight (BMI < 18.5 kg/m^2^), normal (18.5 ≤ BMI < 24.0 kg/m^2^), overweight (24.0 ≤ BMI < 28.0 kg/m^2^) and obese (BMI ≥ 28.0 kg/m^2^)^38^.

### Statistical analysis

We described the characteristics of the cross-section subjects using means and 95%CI for continuous variables and percentages for categorical variables. The continuous variables were compared by t test and the categorical variables were compared by Rao-Scott-χ^2^ test. We used QR models to explore the associations of obesity and smoking in different quantiles of BMI (or WC) (P_10_, P_20_, P_30_, P_50_, P_70_, P_85_, P_95_) by genders. QR model has high flexibility for modeling data with heterogeneous conditional distributions, which matched our study very well. In addition, the complex sampling analysis was used in this study in order to generate representative estimates of the associations. Statistical analyses were performed by R version 3.3.3, using the package “quantreg”^39^ with the statement “WEIGHT”^40^. Statistical significance was set at *p* < 0.05.

### Ethics Approval

This study conformed to the provisions of the Declaration of Helsinki (as revised in Fortaleza, Brazil, October 2013). It was approved by the Ethics Committee of Jilin University School of Public Health (reference number 2012-R-011) and the Bureau of Public Health of Jilin Province (Reference Number: 2012–10). All participants provided written informed consent and all methods were performed in accordance with the relevant guidelines and regulations.

## Supplementary information

Supplementary Material to: The associations between smoking and obesity in northeast China: a quantile regression analysis

## Data Availability

The survey was implemented by School of Public Health, Jilin University and Jilin Center for Disease Control and Prevention in Jilin Province in 2012. According to relevant regulations, we were sorry that the data can’t be shared.
