# Supplementary material for: The associations between smoking and obesity in northeast China: a quantile regression analysis
Source: Sci Rep. 2019 Mar 14;9:3732. doi: 10.1038/s41598-019-39425-6 (PMC6418137; doi:10.1038/s41598-019-39425-6)
Supplement: Supplementary file 1 — Supplementary Material to: The associations between smoking and obesity in northeast China: a quantile regression analysis [file 41598_2019_39425_MOESM1_ESM.docx]

**Supplementary Material to:**

**The associations between** **smoking and obesity in northeast China:**

**a quantile regression analysis**

Mengzi Sun^a^, Yan Jiang^b^, Chong Sun^a^, Jiagen Li^a^, Xin Guo^a^, Yaogai Lv^a^, Yaqin Yu^a^,

Yan Yao^a,^*, Lina Jin^a,^*

^a^ Department of Epidemiology and Biostatistics, School of Public Health, Jilin University, Changchun, Jilin, China

^b^ Department of Biostatistics, Graduate School of Public Health, University of Pittsburgh, PA, USA

* Correspondence: [jinln@jlu.edu.cn](mailto:jinln@jlu.edu.cn); Tel.: +86-431-85619451;

[yaoyan@jlu.edu.cn](mailto:yaoyan@jlu.edu.cn); Tel.: +86-431-85619456;

**Sampling Method**

Five-stage stratified random cluster sampling was used to select the study sample. In the first stage, 32 districts/counties were identified in proportion to population, geographic location and ethnicity, form 9 cities (Changchun, Jilin, Siping, Liaoyuan, Tonghua, Baishan, Songyuan, Baicheng and Yanbian). At the second stage, three or four towns (depending on the size of the district) were selected by stratified random sampling to guarantee the representativeness of each sample. In the third stage, three neighborhood committees were chosen by stratified random sampling from each of the towns previously selected. In the fourth stage, one village from each chosen neighborhood committee was selected by simple random sampling. In the final stage, cluster random sampling was used to identify individuals aged 18 to 79 years old from each of the villages selected for the study.

A strict quality control system was implemented at each stage of the data collection to ensure uniformity and accuracy of the data. Before the formal investigation, a pre-investigation was conducted to explore the design of the questionnaire. In addition, systematic training for all investigators was organized to teach them on how to administer the screening questionnaire, and how to take anthropometric measurements. Before the interview, the identity of each participant was confirmed by the investigator. The validity of each answered questionnaire was examined by the interviewer after the participant had completed the questionnaire in order to ascertain whether the responses were consistent with the real situation. After the fieldwork, all data were processed by parallel double entry. Three verifications were carried out to check for incomplete or inconsistent responses, and then deleted the missing data that cannot be repaired.

The data collection included an intensive investigation and household survey. The purpose of the investigation was explained to the participants before the interview, and then given the option to sign an informed consent form, and only those who consented to participate in the study proceeded to answering the questions and doing the further investigations. The study was approved by the Institutional Review Board of the School of Public Health, Jilin University, Jilin, China. The demographic information collected included gender, age, family income, level of education, occupation, smoking, drinking, exercise, diagnosis and treatment of diabetes, and self-reported family history of diabetes. In addition, anthropometric measurements including height, weight, and fasting blood sugar were taken.

Table S1 Quantiles of WC in different BMI groups by smoking status in males.

| Quantile | Total | |  | Underweight | | | |  | Normal | | | |  | Overweight | | | |  | Obese | | | |
| --- | --- | --- | --- | --- | --- | --- | --- | --- | --- | --- | --- | --- | --- | --- | --- | --- | --- | --- | --- | --- | --- | --- |
| (%) | Y* | N* |  | Y | N | Z | *p* |  | Y | N | Z | *p* |  | Y | N | Z | *p* |  | Y | N | Z | *p* |
| 10 | 70.0 | 72.0 |  | 62.0 | 61.2 | 0.944 | 0.331 |  | 69.0 | 70.0 | 14.916 | <0.001 |  | 81.8 | 81.4 | 0.584 | 0.445 |  | 91.2 | 90.9 | 0.823 | 0.364 |
| 20 | 73.8 | 76.8 |  | 63.8 | 63.8 |  |  |  | 71.9 | 72.8 |  |  |  | 84.0 | 83.8 |  |  |  | 93.2 | 93.2 |  |  |
| 30 | 77.0 | 80.0 |  | 65.0 | 64.9 |  |  |  | 73.8 | 74.6 |  |  |  | 85.9 | 85.6 |  |  |  | 95.2 | 95.2 |  |  |
| 50 | 83.0 | 85.9 |  | 67.0 | 67.0 |  |  |  | 77.0 | 77.8 |  |  |  | 88.9 | 88.8 |  |  |  | 98.9 | 98.2 |  |  |
| 75 | 90.5 | 92.8 |  | 71.0 | 69.4 |  |  |  | 81.8 | 82.7 |  |  |  | 92.7 | 92.4 |  |  |  | 103.2 | 102.8 |  |  |
| 85 | 94.8 | 96.8 |  | 72.6 | 71.4 |  |  |  | 84.0 | 85.0 |  |  |  | 94.9 | 94.5 |  |  |  | 106.1 | 105.8 |  |  |
| 95 | 101.0 | 102.8 |  | 76.0 | 75.0 |  |  |  | 88.6 | 89.0 |  |  |  | 98.2 | 98.6 |  |  |  | 112.0 | 109.5 |  |  |

^*^Y for smokers, N for non-smokers.

Underweight: BMI < 18.5 kg/m^2^; Normal: 18.5 ≤ BMI < 24.0 kg/m^2^; Overweight: 24.0 ≤ BMI < 28.0 kg/m^2^; Obese: BMI ≥ 28.0 kg/m^2^.

Numbers in the Table are WC at different quantiles

Table S2 Quantiles of WC for different BMI groups by smoking status in females.

| Quantile | Total | |  | Underweight | | | |  | Normal | | | |  | Overweight | | | |  | Obese | | | |
| --- | --- | --- | --- | --- | --- | --- | --- | --- | --- | --- | --- | --- | --- | --- | --- | --- | --- | --- | --- | --- | --- | --- |
| (%) | Y* | N* |  | Y | N | Z | *p* |  | Y | N | Z | *p* |  | Y | N | Z | *p* |  | Y | N | Z | *p* |
| 10 | 67.8 | 67.3 |  | 60.2 | 58.5 | 22.67 | <0.001 |  | 67.4 | 66.0 | 25.592 | <0.001 |  | 78.6 | 76.8 | 24.764 | <0.001 |  | 87.0 | 84.8 | 4.733 | 0.03 |
| 20 | 71.6 | 71.0 |  | 62.0 | 60.2 |  |  |  | 69.8 | 68.4 |  |  |  | 80.5 | 79.0 |  |  |  | 89.6 | 87.9 |  |  |
| 30 | 74.4 | 74.1 |  | 63.5 | 61.4 |  |  |  | 72.3 | 70.4 |  |  |  | 82.5 | 81.0 |  |  |  | 91.5 | 89.9 |  |  |
| 50 | 80.0 | 79.9 |  | 66.5 | 63.4 |  |  |  | 75.8 | 73.8 |  |  |  | 85.9 | 84.0 |  |  |  | 94.8 | 93.4 |  |  |
| 75 | 87.8 | 87.0 |  | 70.5 | 67.0 |  |  |  | 80.0 | 78.5 |  |  |  | 91.0 | 88.5 |  |  |  | 99.5 | 98.0 |  |  |
| 85 | 92.0 | 90.9 |  | 72.5 | 69.2 |  |  |  | 82.8 | 81.0 |  |  |  | 93.0 | 90.9 |  |  |  | 102.0 | 101.0 |  |  |
| 95 | 97.0 | 97.2 |  | 75.2 | 73.4 |  |  |  | 86.4 | 84.8 |  |  |  | 96.8 | 94.5 |  |  |  | 107.3 | 106.5 |  |  |

^*^Y for smokers, N for non-smokers.

Underweight: BMI < 18.5 kg/m^2^; Normal: 18.5 ≤ BMI < 24.0 kg/m^2^; Overweight: 24.0 ≤ BMI < 28.0 kg/m^2^; Obese: BMI ≥ 28.0 kg/m^2^.

Numbers in the Table are WC at different quantiles

Table S3 Coefficients and *p*-value in QR models for independents for males in Model 1.

| Variables | | P_10_ | | P_20_ | | P_30_ | | P_50_ | | P_70_ | | P_85_ | | P_95_ | |
| --- | --- | --- | --- | --- | --- | --- | --- | --- | --- | --- | --- | --- | --- | --- | --- |
|  | | β | *p* | β | *p* | β | *p* | β | *p* | β | *p* | β | *p* | β | *p* |
| AGE | | 0.018 | 0.003 | 0.026 | <0.001 | 0.016 | 0.007 | 0.010 | 0.152 | 0.000 | 0.947 | -0.017 | 0.061 | -0.034 | 0.003 |
| Urban-country | | 0.118 | 0.493 | 0.272 | 0.111 | 0.443 | <0.001 | 0.355 | 0.017 | 0.344 | 0.018 | 0.237 | 0.161 | 0.169 | 0.582 |
| Educational level | |  |  |  |  |  |  |  |  |  |  |  |  |  |  |
| Junior high school | 0.298 | 0.183 | 0.176 | 0.387 | 0.306 | 0.096 | 0.708 | <0.001 | 0.494 | 0.005 | 0.733 | 0.001 | 0.656 | 0.077 |  |
| High school | 0.372 | 0.210 | 0.442 | 0.059 | 0.420 | 0.050 | 0.649 | 0.002 | 0.567 | 0.004 | 0.736 | 0.001 | 0.657 | 0.109 |  |
| College degree or above | 0.607 | 0.035 | 0.852 | 0.007 | 0.953 | <0.001 | 1.441 | <0.001 | 1.088 | <0.001 | 1.055 | 0.002 | 0.601 | 0.207 |  |
| Marital Status | |  |  |  |  |  |  |  |  |  |  |  |  |  |  |
| Single | -1.715 | <0.001 | -1.705 | <0.001 | -2.003 | <0.001 | -2.198 | <0.001 | -1.862 | <0.001 | -1.061 | 0.014 | -0.026 | 0.958 |  |
| Divorce or separation | 0.497 | 0.219 | 0.430 | 0.168 | 0.123 | 0.729 | -0.009 | 0.981 | -0.231 | 0.591 | -0.464 | 0.282 | -0.996 | 0.416 |  |
| Widowed | -0.474 | 0.087 | -1.108 | <0.001 | -1.033 | 0.004 | -0.825 | 0.020 | -1.270 | <0.001 | -1.326 | 0.005 | -1.184 | 0.060 |  |
| Current smoking | | -0.905 | <0.001 | -0.856 | <0.001 | -0.971 | <0.001 | -0.928 | <0.001 | -0.774 | <0.001 | -1.005 | <0.001 | -0.970 | <0.001 |
| Drinking | | 0.432 | <0.001 | 0.488 | <0.001 | 0.452 | <0.001 | 0.181 | 0.206 | 0.178 | 0.276 | -0.203 | 0.261 | -0.336 | 0.229 |
| Diet Habits | |  |  |  |  |  |  |  |  |  |  |  |  |  |  |
| Meat more | 0.514 | 0.017 | 0.606 | <0.001 | 0.778 | <0.001 | 0.753 | <0.001 | 0.588 | <0.001 | 0.544 | 0.048 | 0.481 | 0.263 |  |
| Vegetable more | -0.309 | 0.036 | -0.453 | <0.001 | -0.499 | <0.001 | 0.527 | <0.001 | -0.403 | 0.018 | -0.805 | <0.001 | -0.986 | <0.001 |  |
| Physical Exercise | |  |  |  |  |  |  |  |  |  |  |  |  |  |  |
| Sometimes | -0.019 | 0.921 | 0.171 | 0.383 | 0.108 | 0.518 | 0.167 | 0.384 | 0.455 | 0.028 | 0.439 | 0.048 | 0.774 | 0.036 |  |
| Never or rare | -0.177 | 0.306 | -0.247 | 0.169 | -0.326 | 0.028 | -0.346 | 0.019 | -0.067 | 0.686 | -0.149 | 0.442 | -0.088 | 0.786 |  |
| Occupation | |  |  |  |  |  |  |  |  |  |  |  |  |  |  |
| Mental work | 0.065 | 0.795 | 0.253 | 0.288 | 0.195 | 0.257 | 0.180 | 0.385 | 0.319 | 0.101 | 0.078 | 0.753 | -0.098 | 0.756 |  |
| Non-physical or non-mental work | -0.248 | 0.493 | -0.194 | 0.399 | -0.129 | 0.507 | -0.176 | 0.435 | 0.062 | 0.784 | 0.334 | 0.231 | 0.251 | 0.421 |  |

Table S4 Coefficients and *p*-value in QR models for independents for females in Model 1.

| Variables | | | | P_10_ | | | | | P_20_ | | | | | P_30_ | | | | P_50_ | | | | P_70_ | | | | | P_85_ | | | | | P_95_ | |
| --- | --- | --- | --- | --- | --- | --- | --- | --- | --- | --- | --- | --- | --- | --- | --- | --- | --- | --- | --- | --- | --- | --- | --- | --- | --- | --- | --- | --- | --- | --- | --- | --- | --- |
|  | | β | | | *p* | | β | | | *p* | | β | | | *p* | | β | | *p* | | β | | | *p* | | β | | | *p* | | β | | *p* |
| AGE | | 0.048 | | | <0.001 | | 0.060 | | | <0.001 | | 0.068 | | | <0.001 | | 0.069 | | <0.001 | | 0.074 | | | <0.001 | | 0.060 | | | <0.001 | | 0.023 | | 0.184 |
| Urban-country | | 0.133 | | | 0.393 | | -0.083 | | | 0.560 | | -0.080 | | | 0.517 | | -0.476 | | <0.001 | | -0.484 | | | 0.003 | | -0.214 | | | 0.259 | | -0.115 | | 0.710 |
| Educational level | | |  | | |  | |  | | |  | |  | | |  | |  | |  | | |  | |  | | |  | |  | | |  |
| Junior high school | -0.194 | | | 0.347 | | -0.039 | | | 0.796 | | -0.199 | | | 0.262 | | 0.083 | | 0.547 | | -0.076 | | | 0.658 | | -0.282 | | | 0.286 | | -0.335 | | 0.323 |  |
| High school | -0.152 | | | 0.503 | | -0.127 | | | 0.453 | | -0.316 | | | 0.104 | | -0.312 | | 0.056 | | -0.326 | | | 0.114 | | -0.500 | | | 0.054 | | -0.823 | | 0.035 |  |
| College degree or above | -0.244 | | | 0.389 | | -0.404 | | | 0.068 | | -0.572 | | | 0.024 | | -0.713 | | 0.002 | | -0.676 | | | 0.003 | | -1.103 | | | 0.001 | | -2.043 | | <0.001 |  |
| Marital Status | | |  | | |  | |  | | |  | |  | | |  | |  | |  | | |  | |  | | |  | |  | | |  |
| Single | -1.122 | | | <0.001 | | -1.418 | | | <0.001 | | -0.994 | | | <0.001 | | -0.997 | | <0.001 | | -1.549 | | | <0.001 | | -2.244 | | | <0.001 | | -1.697 | | 0.032 |  |
| Divorce or separation | -0.051 | | | 0.890 | | -0.089 | | | 0.809 | | 0.006 | | | 0.984 | | 0.027 | | 0.942 | | 0.052 | | | 0.921 | | 0.185 | | | 0.775 | | -0.557 | | 0.536 |  |
| Widowed | -0.356 | | | 0.232 | | -0.485 | | | 0.021 | | -0.723 | | | <0.001 | | -0.566 | | 0.019 | | -0.766 | | | 0.002 | | -0.643 | | | 0.158 | | -0.012 | | 0.979 |  |
| Current smoking | | -1.751 | | | <0.001 | | -1.596 | | | <0.001 | | -1.399 | | | <0.001 | | -1.170 | | <0.001 | | -0.807 | | | 0.001 | | -0.633 | | | 0.049 | | -0.211 | | 0.670 |
| Drinking | | 0.709 | | | 0.007 | | 0.586 | | | <0.001 | | 0.637 | | | <0.001 | | 0.627 | | <0.001 | | 0.268 | | | 0.274 | | 0.414 | | | 0.155 | | -0.098 | | 0.815 |
| Diet Habits | |  | | |  | |  | | |  | |  | | |  | |  | |  | |  | | |  | |  | | |  | |  | |  |
| Meat more | 0.023 | | | 0.931 | | 0.247 | | | 0.422 | | 0.283 | | | 0.188 | | 0.383 | | 0.275 | | 0.727 | | | 0.038 | | 0.592 | | | 0.179 | | 0.022 | | 0.967 |  |
| Vegetable more | -0.295 | | | 0.030 | | -0.209 | | | 0.071 | | -0.149 | | | 0.199 | | 0.242 | | 0.036 | | -0.288 | | | 0.024 | | -0.436 | | | 0.016 | | -0.378 | | 0.194 |  |
| Physical Exercise | | |  | | |  | |  | | |  | |  | | |  | |  | |  | | |  | |  | | |  | |  | | |  |
| Sometimes | -0.678 | | | <0.001 | | -0.561 | | | 0.001 | | -0.588 | | | <0.001 | | -0.466 | | 0.004 | | -0.074 | | | 0.709 | | -0.116 | | | 0.697 | | -0.171 | | 0.671 |  |
| Never or rare | -0.513 | | | 0.006 | | -0.482 | | | 0.001 | | -0.438 | | | <0.001 | | -0.485 | | <0.001 | | -0.260 | | | 0.078 | | -0.279 | | | 0.228 | | -0.275 | | 0.455 |  |
| Occupation | |  | | |  | |  | | |  | |  | | |  | |  | |  | |  | | |  | |  | | |  | |  | |  |
| Mental work | -0.303 | | | 0.120 | | -0.239 | | | 0.127 | | -0.267 | | | 0.076 | | -0.201 | | 0.288 | | -0.136 | | | 0.471 | | -.445 | | | -0.106 | | -0.573 | | 0.224 |  |
| Non-physical or non-mental work | 0.012 | | | 0.949 | | 0.010 | | | 0.947 | | -0.005 | | | 0.975 | | 0.191 | | 0.192 | | 0.213 | | | 0.165 | | 0.155 | | | 0.499 | | 0.040 | | 0.889 |  |

Table S5 Coefficients and *p*-value in QR models for independents for males in Model 2.

| Variables | | | P_10_ | | | P_20_ | | | P_30_ | | | P_50_ | | | P_70_ | | | P_85_ | | | P_95_ | |
| --- | --- | --- | --- | --- | --- | --- | --- | --- | --- | --- | --- | --- | --- | --- | --- | --- | --- | --- | --- | --- | --- | --- |
|  | | β | | *p* | β | | *p* | β | | *p* | β | | *p* | β | | *p* | β | | *p* | β | | *p* |
| AGE | | 0.129 | | <0.001 | 0.143 | | <0.001 | 0.153 | | <0.001 | 0.129 | | <0.001 | 0.109 | | <0.001 | 0.049 | | 0.041 | 0.067 | | 0.037 |
| Urban-country | | 0.881 | | 0.044 | 0.857 | | 0.069 | 1.338 | | 0.001 | 1.059 | | 0.010 | 0.907 | | 0.026 | 0.534 | | 0.352 | -0.635 | | 0. 316 |
| Educational level | |  | |  |  | |  |  | |  |  | |  |  | |  |  | |  |  | |  |
| Junior high school | 0.759 | | 0.258 | 1.357 | | 0.013 | 1.883 | | <0.001 | 2.340 | | <0.001 | 2.694 | | <0.001 | 2.167 | | 0.005 | 1.609 | | 0.073 |  |
| High school | 1.896 | | 0.016 | 2.714 | | <0.001 | 2.645 | | <0.001 | 2.794 | | <0.001 | 3.296 | | <0.001 | 2.075 | | 0.006 | 1.650 | | 0.094 |  |
| College degree or above | 2.919 | | 0.001 | 4.071 | | <0.001 | 4.443 | | <0.001 | 4.798 | | <0.001 | 4.656 | | <0.001 | 3.199 | | 0.001 | 2.406 | | 0.027 |  |
| Marital Status | |  | |  |  | |  |  | |  |  | |  |  | |  |  | |  |  | |  |
| Single | -3.769 | | <0.001 | -4.428 | | <0.001 | -5.109 | | <0.001 | -5.5126 | | <0.001 | -5.022 | | <0.001 | -4.269 | | <0.001 | -1.385 | | 0.286 |  |
| Divorce or separation | -0.793 | | 0.402 | -1.228 | | 0.356 | -0.330 | | 0.769 | 0.733 | | 0.525 | -1.186 | | 0.333 | -0.764 | | 0.587 | -1.010 | | 0.670 |  |
| Widowed | -1.388 | | 0.084 | -2.900 | | 0.025 | -2.253 | | 0.064 | -3.175 | | <0.001 | -3.931 | | 0.001 | -2.596 | | 0.089 | -5.247 | | <0.001 |  |
| Current smoking | | -1.836 | | <0.001 | -2.071 | | <0.001 | -1.907 | | <0.001 | -1.512 | | <0.001 | -1.573 | | <0.001 | -1.540 | | 0.005 | -1.609 | | 0.015 |
| Drinking | | 1.641 | | <0.001 | 1.714 | | <0.001 | 1.771 | | <0.001 | 1.217 | | 0.002 | 1.316 | | 0.007 | -0.144 | | 0.763 | -0.141 | | 0.826 |
| Diet Habits | |  | |  |  | |  |  | |  |  | |  |  | |  |  | |  |  | |  |
| Meat more | 1.754 | | 0.007 | 2.714 | | <0.001 | 2.454 | | <0.001 | 2.245 | | <0.001 | 3.045 | | <0.001 | 1.548 | | 0.045 | 1.349 | | 0.164 |  |
| Vegetable more | -0.578 | | 0.209 | -1.000 | | 0.024 | -1.705 | | <0.001 | -1.444 | | <0.001 | -1.402 | | 0.001 | -2.588 | | <0.001 | -3.371 | | <0.001 |  |
| Physical Exercise | |  | |  |  | |  |  | |  |  | |  |  | |  |  | |  |  | |  |
| Sometimes | 0.125 | | 0.828 | 0.357 | | 0.566 | 0.488 | | 0.347 | 0.629 | | 0.223 | 0.886 | | 0.071 | 0.655 | | 0.389 | 1.277 | | 0.120 |  |
| Never or rare | -0.579 | | 0.296 | -0.714 | | 0.195 | -0.910 | | 0.048 | 0.524 | | 0.241 | -0.439 | | 0.278 | -0.472 | | 0.403 | -1.337 | | 0.073 |  |
| Occupation | |  | |  |  | |  |  | |  |  | |  |  | |  |  | |  |  | |  |
| Mental work | 1.086 | | 0.099 | 1.429 | | 0.026 | 1.564 | | 0.001 | 1.963 | | 0.001 | 1.349 | | 0.009 | 1.489 | | 0.029 | -0.104 | | 0.895 |  |
| Non-physical or non-mental work | -0.319 | | 0.651 | 0.286 | | 0.684 | 0.148 | | 0.776 | 0.271 | | 0.666 | 0.929 | | 0.151 | 1.144 | | 0.050 | 0.631 | | 0.504 |  |

Table S6 Coefficients and *p*-value in QR models for independents for females in Model 2.

| Variables | | | | P_10_ | | | | | P_20_ | | | | | P_30_ | | | | P_50_ | | | | P_70_ | | | | | P_85_ | | | | | P_95_ | | |
| --- | --- | --- | --- | --- | --- | --- | --- | --- | --- | --- | --- | --- | --- | --- | --- | --- | --- | --- | --- | --- | --- | --- | --- | --- | --- | --- | --- | --- | --- | --- | --- | --- | --- | --- |
|  | | β | | | *p* | | β | | | *p* | | β | | | *p* | | β | | *p* | | β | | | *p* | | β | | | *p* | | β | | | *p* |
| AGE | | 0.249 | | | <0.001 | | 0.273 | | | <0.001 | | 0.295 | | | <0.001 | | 0.300 | | <0.001 | | 0.314 | | | <0.001 | | 0.301 | | | <0.001 | | 0.288 | | | <0.001 |
| Urban-country | | -0.763 | | | 0.0537 | | -1.154 | | | 0.001 | | -1.416 | | | <0.001 | | -1.200 | | <0.001 | | -1.000 | | | <0.001 | | -1.394 | | | 0.002 | | -2.208 | | | 0.008 |
| Educational level | | |  | | |  | |  | | |  | |  | | |  | |  | |  | | |  | |  | | |  | |  | | |  | |
| Junior high school | -0.086 | | | 0.866 | | -0.438 | | | 0.304 | | -0.594 | | | 0.183 | | -0.600 | | 0.147 | | -0.971 | | | 0.066 | | -1.183 | | | 0.075 | | -0.834 | | | 0.326 |  |
| High school | -0.603 | | | 0.319 | | 0.883 | | | 0.071 | | -1.065 | | | 0.037 | | -1.500 | | <0.001 | | -1.385 | | | 0.005 | | -1.465 | | | 0.022 | | -0.551 | | | 0.622 |  |
| College degree or above | -0.326 | | | 0.650 | | -0.990 | | | 0.051 | | -1.154 | | | 0.068 | | -2.100 | | <0.001 | | -1.886 | | | 0.021 | | -2.043 | | | 0.030 | | -2.760 | | | 0.0718 |  |
| Marital Status | | |  | | |  | |  | | |  | |  | | |  | |  | |  | | |  | |  | | |  | |  | | |  | |
| Single | -1.552 | | | 0.014 | | -2.192 | | | <0.001 | | -3.138 | | | <0.001 | | -2.000 | | 0.011 | | -2.971 | | | <0.001 | | -3.479 | | | 0.003 | | 0.334 | | | 0.893 |  |
| Divorce or separation | -1.184 | | | 0.290 | | -0.452 | | | 0.621 | | -0.167 | | | 0.894 | | -0.100 | | 0.899 | | -0.886 | | | 0.419 | | 0.014 | | | 0.994 | | 2.100 | | | 0.374 |  |
| Widowed | -0.128 | | | 0.867 | | -1.059 | | | 0.052 | | -1.884 | | | 0.003 | | -2.300 | | 0.001 | | -1.629 | | | 0.029 | | -1.508 | | | 0.074 | | -0.645 | | | 0.643 |  |
| Current smoking | | -2.765 | | | <0.001 | | -3.223 | | | <0.001 | | -2.458 | | | <0.001 | | -2.000 | | 0.001 | | -1.000 | | | 0.153 | | -0.315 | | | 0.650 | | 0.108 | | | 0.912 |
| Drinking | | 1.663 | | | 0.001 | | 1.650 | | | 0.001 | | 1.694 | | | <0.001 | | 0.500 | | 0.315 | | 0.743 | | | 0.253 | | 1.194 | | | 0.112 | | 0.400 | | | 0.671 |
| Diet Habits | |  | | |  | |  | | |  | |  | | |  | |  | |  | |  | | |  | |  | | |  | |  | | |  |
| Meat more | -0.495 | | | 0.631 | | 0.694 | | | 0.347 | | 1.301 | | | 0.102 | | 1.100 | | 0.155 | | 1.300 | | | 0.162 | | 1.926 | | | 0.232 | | 2.297 | | | 0.078 |  |
| Vegetable more | -0.969 | | | 0.002 | | -0.957 | | | 0.001 | | -0.898 | | | 0.001 | | -0.400 | | 0.203 | | -0.843 | | | 0.013 | | -1.232 | | | 0.004 | | -1.388 | | | 0.055 |  |
| Physical Exercise | | |  | | |  | |  | | |  | |  | | |  | |  | |  | | |  | |  | | |  | |  | | |  | |
| Sometimes | -0.708 | | | 0.213 | | -0.665 | | | 0.116 | | -1.013 | | | 0.007 | | -1.100 | | 0.012 | | -0.457 | | | 0.344 | | -0.329 | | | 0.557 | | -0.185 | | | 0.857 |  |
| Never or rare | -1.329 | | | 0.006 | | -0.879 | | | 0.032 | | -1.056 | | | 0.005 | | -1.400 | | <0.001 | | -0.586 | | | 0.186 | | -0.523 | | | 0.294 | | -1.102 | | | 0.247 |  |
| Occupation | |  | | |  | |  | | |  | |  | | |  | |  | |  | |  | | |  | |  | | |  | |  | | |  |
| Mental work | -0.403 | | | 0.334 | | 0.019 | | | 0.962 | | -0.047 | | | 0.909 | | -0.400 | | 0.437 | | -0.714 | | | 0.212 | | - 1.515 | | | 0.022 | | -1.694 | | | 0.180 |  |
| Non-physical or non-mental work | 0.446 | | | 0.345 | | 1.336 | | | 0.001 | | 1.456 | | | <0.001 | | 1.200 | | 0.004 | | 0.943 | | | 0.027 | | 0.613 | | | 0.263 | | 0.611 | | | 0.469 |  |

Table S7 Coefficients and *p*-value in QR models for independents for males in Model 3.

| Variables | | | P_10_ | | | P_20_ | | | P_30_ | | | P_50_ | | | P_70_ | | | P_85_ | | | P_95_ | |
| --- | --- | --- | --- | --- | --- | --- | --- | --- | --- | --- | --- | --- | --- | --- | --- | --- | --- | --- | --- | --- | --- | --- |
|  | | β | | *p* | β | | *p* | β | | *p* | β | | *p* | β | | *p* | β | | *p* | β | | *p* |
| BMI | | 2.373 | | <0.001 | 2.444 | | <0.001 | 2.472 | | <0.001 | 2.503 | | <0.001 | 2.507 | | <0.001 | 2.545 | | <0.001 | 2.631 | | <0.001 |
| AGE | | 0.065 | | <0.001 | 0.080 | | <0.001 | 0.087 | | <0.001 | 0.089 | | <0.001 | 0.107 | | <0.001 | 0.128 | | <0.001 | 0.151 | | <0.001 |
| Urban-country | | -0.013 | | 0.958 | 0.150 | | 0.369 | 0.203 | | 0.256 | 0.070 | | 0.703 | 0.018 | | 0.913 | 0.003 | | 0.987 | 0.273 | | 0.339 |
| Educational level | |  | |  |  | |  |  | |  |  | |  |  | |  |  | |  |  | |  |
| Junior high school | 0.505 | | 0.123 | 0.598 | | 0.016 | 0.814 | | <0.001 | 0.727 | | 0.006 | 0.949 | | <0.001 | 0.897 | | 0.003 | 0.708 | | 0.106 |  |
| High school | 0.848 | | 0.007 | 0.859 | | 0.001 | 1.105 | | <0.001 | 0.985 | | <0.001 | 1.270 | | <0.001 | 1.105 | | 0.001 | 1.256 | | 0.012 |  |
| College degree or above | 1.278 | | 0.004 | 1.458 | | <0.001 | 1.517 | | <0.001 | 1.561 | | <0.001 | 1.778 | | <0.001 | 1.764 | | <0.001 | 1.669 | | 0.002 |  |
| Marital Status | |  | |  |  | |  |  | |  |  | |  |  | |  |  | |  |  | |  |
| Single | -0.317 | | 0.407 | -0.613 | | 0.828 | -0.477 | | 0.181 | -0.859 | | 0.004 | -0.566 | | 0.079 | -0.170 | | 0.612 | 0.077 | | 0.876 |  |
| Divorce or separation | -1.125 | | 0.118 | 0.132 | | 0.874 | -0.435 | | 0.360 | -0.158 | | 0.757 | 0.765 | | 0.316 | 1.040 | | 0.154 | 0.278 | | 0.675 |  |
| Widowed | -0.552 | | 0.312 | -0.184 | | 0.777 | -0.672 | | 0.113 | -0.524 | | 0.447 | -0.858 | | 0.063 | -0.793 | | 0.386 | -0.778 | | 0.238 |  |
| Current smoking | | 0.547 | | 0.014 | 0.645 | | 0.001 | 0.603 | | 0.001 | 0.623 | | <0.001 | 0.325 | | 0.080 | 0.479 | | 0.028 | 1.068 | | 0.001 |
| Drinking | | 0.520 | | 0.016 | 0.675 | | 0.001 | 0.641 | | 0.001 | 0.519 | | 0.002 | 0.242 | | 0.181 | 0.546 | | 0.006 | 0.241 | | 0.437 |
| Diet Habits | |  | |  |  | |  |  | |  |  | |  |  | |  |  | |  |  | |  |
| Meat more | 0.354 | | 0.310 | 0.610 | | 0.046 | 0.753 | | 0.001 | 0.768 | | 0.001 | 0.981 | | <0.001 | 0.931 | | 0.003 | 1.590 | | <0.001 |  |
| Vegetable more | -0.284 | | 0.253 | -0.325 | | -0.166 | -0.242 | | 0.249 | -0.407 | | 0.017 | -0.565 | | 0.005 | -0.026 | | 0.911 | -0.025 | | 0.935 |  |
| Physical Exercise | |  | |  |  | |  |  | |  |  | |  |  | |  |  | |  |  | |  |
| Sometimes | -0.049 | | 0.874 | 0.015 | | 0.046 | 0.047 | | 0.835 | -0.029 | | 0.886 | 0.000 | | 0.998 | 0.143 | | 0.627 | -0.207 | | 0.552 |  |
| Never or rare | -0.121 | | 0.661 | -0.100 | | -0.166 | -0.166 | | 0.454 | -0.194 | | 0.278 | -0.110 | | 0.589 | -0.109 | | 0.681 | 0.017 | | 0.962 |  |
| Occupation | |  | |  |  | |  |  | |  |  | |  |  | |  |  | |  |  | |  |
| Mental work | 1.331 | | <0.001 | 1.122 | | <0.001 | 1.187 | | <0.001 | 1.023 | | <0.001 | 0.879 | | <0.001 | 1.212 | | <0.001 | 0.812 | | 0.024 |  |
| Non-physical or non-mental work | 1.157 | | <0.001 | 0.915 | | <0.001 | 0.728 | | 0.001 | 0.814 | | 0.002 | 0.387 | | 0.087 | 0.211 | | 0.591 | 0.204 | | 0.620 |  |

Table S8 Coefficients and *p*-value in QR models for independents for females in Model 3.

| Variables | | | | P_10_ | | | | | P_20_ | | | | | P_30_ | | | | P_50_ | | | | P_70_ | | | | | P_85_ | | | | | P_95_ | | |
| --- | --- | --- | --- | --- | --- | --- | --- | --- | --- | --- | --- | --- | --- | --- | --- | --- | --- | --- | --- | --- | --- | --- | --- | --- | --- | --- | --- | --- | --- | --- | --- | --- | --- | --- |
|  | | β | | | *p* | | β | | | *p* | | β | | | *p* | | β | | *p* | | β | | | *p* | | β | | | *p* | | β | | | *p* |
| BMI | | 2.020 | | | <0.001 | | 2.100 | | | <0.001 | | 2.168 | | | <0.001 | | 2.206 | | <0.001 | | 2.234 | | | <0.001 | | 2.283 | | | <0.001 | | 2.307 | | | <0.001 |
| AGE | | 0.130 | | | <0.001 | | 0.142 | | | <0.001 | | 0.153 | | | <0.001 | | 0.153 | | <0.001 | | 0.151 | | | <0.001 | | 0.156 | | | <0.001 | | 0.161 | | | <0.001 |
| Urban-country | | -1.111 | | | <0.001 | | -0.798 | | | <0.001 | | -0.918 | | | <0.001 | | -0.711 | | 0.001 | | -0.498 | | | 0.031 | | -0.475 | | | 0.143 | | -0.251 | | | 0.553 |
| Educational level | | |  | | |  | |  | | |  | |  | | |  | |  | |  | | |  | |  | | |  | |  | | |  | |
| Junior high school | 0.045 | | | 0.857 | | -0.238 | | | 0.241 | | -0.196 | | | 0.355 | | -0.429 | | 0.115 | | -0.494 | | | 0.055 | | -0.317 | | | 0.315 | | -0.641 | | | 0.111 |  |
| High school | -0.315 | | | 0.325 | | -0.569 | | | 0.021 | | -0.343 | | | 0.144 | | -0.674 | | 0.012 | | -0.704 | | | 0.023 | | -0.595 | | | 0.073 | | -0.072 | | | 0.896 |  |
| College degree or above | 0.397 | | | 0.272 | | 0.074 | | | 0.844 | | 0.229 | | | 0.513 | | -0.231 | | 0.544 | | -0.354 | | | 0.445 | | 0.133 | | | 0.921 | | 0.392 | | | 0.632 |  |
| Marital Status | | |  | | |  | |  | | |  | |  | | |  | |  | |  | | |  | |  | | |  | |  | | |  | |
| Single | -0.273 | | | 0.460 | | 0.207 | | | 0.643 | | 0.335 | | | 0.346 | | 0.340 | | 0.489 | | 0.625 | | | 0.225 | | 1.121 | | | 0.182 | | 2.579 | | | 0.054 |  |
| Divorce or separation | -1.035 | | | 0.013 | | -0.701 | | | 0.340 | | 0.204 | | | 0.795 | | 0.110 | | 0.814 | | 0.098 | | | 0.889 | | 0.242 | | | 0.819 | | 0.556 | | | 0.535 |  |
| Widowed | -0.056 | | | 0.906 | | -0.152 | | | 0.646 | | -0.130 | | | 0.661 | | -0.711 | | 0.021 | | -0.425 | | | 0.185 | | -0.255 | | | 0.459 | | 0.037 | | | 0.950 |  |
| Current smoking | | 0.474 | | | 0.113 | | 0.413 | | | 0.111 | | 0.504 | | | 0.090 | | 0.737 | | 0.005 | | 0.975 | | | <0.001 | | 1.039 | | | <0.001 | | 0.956 | | | 0.027 |
| Drinking | | 0.241 | | | 0.373 | | -0.351 | | | 0.208 | | -0.446 | | | 0.101 | | -0.137 | | 0.676 | | 0.025 | | | 0.927 | | -0.250 | | | 0.767 | | -0.376 | | | 0.416 |
| Diet Habits | |  | | |  | |  | | |  | |  | | |  | |  | |  | |  | | |  | |  | | |  | |  | | |  |
| Meat more | 0.769 | | | 0.077 | | 0.263 | | | 0.408 | | -0.055 | | | 0.878 | | 0.123 | | 0.791 | | -0.154 | | | 0.734 | | -0.031 | | | 0.863 | | 0.496 | | | 0.594 |  |
| Vegetable more | -0.182 | | | 0.330 | | -0.425 | | | 0.012 | | -0.504 | | | 0.001 | | -0.188 | | 0.318 | | -0.189 | | | 0.356 | | -0.080 | | | 0.844 | | 0.109 | | | 0.755 |  |
| Physical Exercise | | |  | | |  | |  | | |  | |  | | |  | |  | |  | | |  | |  | | |  | |  | | |  | |
| Sometimes | 0.124 | | | 0.619 | | 0.108 | | | 0.652 | | 0.239 | | | 0.345 | | 0.220 | | 0.348 | | 0.147 | | | 0.555 | | -0.416 | | | 0.441 | | 0.248 | | | 0.604 |  |
| Never or rare | 0.120 | | | 0.597 | | -0.113 | | | 0.602 | | -0.196 | | | 0.308 | | -0.216 | | 0.242 | | -0.232 | | | 0.228 | | -0.611 | | | 0.033 | | 0.118 | | | 0.734 |  |
| Occupation | |  | | |  | |  | | |  | |  | | |  | |  | |  | |  | | |  | |  | | |  | |  | | |  |
| Mental work | 0.359 | | | 0.226 | | -0.028 | | | 0.916 | | -0.018 | | | 0.948 | | 0.305 | | 0.262 | | 0.317 | | | 0.334 | | 0.104 | | | 0.816 | | -0.182 | | | 0.763 |  |
| Non-physical or non-mental work | 0.994 | | | <0.001 | | 0.751 | | | <0.001 | | 0.594 | | | 0.006 | | 0.966 | | <0.001 | | 0.939 | | | <0.001 | | 0.750 | | | 0.008 | | 1.490 | | | 0.001 |  |
